# Development of a comparative genomic fingerprinting assay for rapid and high resolution genotyping of *Arcobacter butzleri*

**DOI:** 10.1186/s12866-015-0426-4

**Published:** 2015-05-07

**Authors:** Andrew L Webb, Peter Kruczkiewicz, L Brent Selinger, G Douglas Inglis, Eduardo N Taboada

**Affiliations:** Agriculture and Agri-Food Canada, 5403 – 1st Avenue S, Lethbridge, AB Canada; Department of Biological Sciences, University of Lethbridge, Lethbridge, AB Canada; Public Health Agency of Canada, Township Rd. 9-1, Lethbridge, AB Canada

**Keywords:** Molecular epidemiology, Subtyping, Comparative genomics, *Arcobacter butzleri*, Genome sequencing

## Abstract

**Background:**

Molecular typing methods are critical for epidemiological investigations, facilitating disease outbreak detection and source identification. Study of the epidemiology of the emerging human pathogen *Arcobacter butzleri* is currently hampered by the lack of a subtyping method that is easily deployable in the context of routine epidemiological surveillance*.* In this study we describe a comparative genomic fingerprinting (CGF) method for high-resolution and high-throughput subtyping of *A. butzleri*. Comparative analysis of the genome sequences of eleven *A. butzleri* strains, including eight strains newly sequenced as part of this project, was employed to identify accessory genes suitable for generating unique genetic fingerprints for high-resolution subtyping based on gene presence or absence within a strain.

**Results:**

A set of eighty-three accessory genes was used to examine the population structure of a dataset comprised of isolates from various sources, including human and non-human animals, sewage, and river water (n=156). A streamlined assay (CGF_40_) based on a subset of 40 genes was subsequently developed through marker optimization. High levels of profile diversity (121 distinct profiles) were observed among the 156 isolates in the dataset, and a high Simpson’s Index of Diversity (ID) observed (ID > 0.969) indicate that the CGF_40_ assay possesses high discriminatory power. At the same time, our observation that 115 isolates in this dataset could be assigned to 29 clades with a profile similarity of 90% or greater indicates that the method can be used to identify clades comprised of genetically similar isolates.

**Conclusions:**

The CGF_40_ assay described herein combines high resolution and repeatability with high throughput for the rapid characterization of *A. butzleri* strains. This assay will facilitate the study of the population structure and epidemiology of *A. butzleri*.

**Electronic supplementary material:**

The online version of this article (doi:10.1186/s12866-015-0426-4) contains supplementary material, which is available to authorized users.

## Background

*Arcobacter butzleri* is closely related to the pathogen *Campylobacter jejuni* [1], and it has been isolated from surface waters, livestock, and animal products [2-5]. The pathogenicity of *A. butzleri* has yet to be resolved [2,6]; although *A. butzleri* has been isolated from the stools of diarrheic human beings, which is highly suggestive of pathogenicity [7-9], it has also been obtained from non-diarrheic individuals, [10,11] suggesting that it is a commensal or that non-pathogenic strains or subtypes exist within the species.

An important facet in the study of pathogens is epidemiology-based analysis of their incidence and distribution. Molecular subtyping or genotyping, which allows the classification of a bacterial species into distinct strains or subtypes based on genetic variation [12,13], forms one of the pillars of molecular epidemiology, through which the identification of etiological agents, patterns of transmission, and potential outbreaks can be carried out with enhanced precision [14]. Until recently, the study of *A. butzleri* has been hampered by the lack of advanced methods for subtyping. A recently developed multi-locus sequence typing (MLST) scheme [15] provides excellent identification of subtypes and has been utilized to examine genetic diversity in *A. butzleri* isolated from people, livestock, and animal products [2,6]. However, this method remains a resource-intensive and relatively low-throughput means of subtyping, which limits the number of isolates that can be analyzed by most research groups [16,17], as evidenced by the relatively small number of isolates that have been contributed to the MLST database for *A. butzleri* by the global research community (*n*=683, PubMLST accessed on October 21, 2014). More importantly, the lack of a highly deployable subtyping method suitable for use in routine surveillance has precluded the large-scale epidemiological surveys required to fully assess the potential role of *A. butzleri* as an emerging pathogen of humans.

Recent advances in sequencing technologies (i.e. next generation sequencing) and bioinformatics have made it possible to rapidly obtain draft whole genome sequence (WGS) data [18] and it is likely that methods based on WGS analysis, including whole-genome MLST (wgMLST), will eventually become the new standard for microbial subtyping in an epidemiological context [19,20]. However, until the resources required for WGS-based subtyping allow it to become practical enough to be deployed in large-scale epidemiological surveillance, there is a continuing need for methods that fulfill performance criteria such as discriminatory power and repeatability, and convenience criteria such as throughput, cost, and ease of use [14]. Recently, Taboada *et al.* [21] employed whole genome analysis to develop a comparative genomic fingerprinting (CGF) method for high-resolution subtyping of *C. jejuni* that was highly concordant with MLST but better suited to large-scale surveillance due to improved throughput and cost relative to MLST. Moreover, by targeting a large number of accessory genes (e.g. 40 loci), the CGF method showed improved discriminatory power compared to MLST, allowing the differentiation of closely related strains with distinct epidemiology [21,22].

The overall goal of the current study was to develop a highly discriminatory CGF assay for *A. butzleri* by employing the strategy described by Taboada *et al.* [21] for *C. jejuni*. Objectives were to: (i) select *A. butzleri* isolates for whole genome sequencing; (ii) utilize whole genome sequence data to identify candidate CGF target genes in the accessory genome; (iii) screen CGF targets against a panel of *A. butzleri* isolates to determine accessory gene frequency and assess accessory genome variability; (iv) select a subset of CGF targets for development of a 40-locus assay (CGF_40_); and (v) evaluate the ability of the CGF_40_ assay to reliably discriminate *A. butzleri* strains. The development of highly deployable genotyping techniques that are suitable for use in routine surveillance will improve our ability to distinguish strains of *A. butzleri* and facilitate the study of its epidemiology.

## Results

### Whole genome sequencing and comparative genomic analysis of *A. butzleri* strains

In order to design a CGF assay for *A. butzleri* it was necessary to perform a comparative genomic analysis of strains representing diverse sources and genetic backgrounds. Twenty-two *A. butzleri* isolates from various sources were genotyped using amplified fragment length polymorphism (AFLP) analysis [23,24] and eight strains representing highly diverse AFLP profiles were chosen for whole-genome sequencing (Additional file 1). The selected strains represented six of thirteen different clades observed in the set of twenty-two isolates analyzed by AFLP.

Illumina 100 bp read paired-end sequencing of *A. but*z*leri* isolates (*n*=8) produced an average of 132 ± 37.0 times coverage based on an assembly size of 2.27 Mbp ± 0.09, with a GC content of 27.3% ± 0.90 and 2.10 ± 1.70 ambiguous bases per 100 kbp. The *de novo* assemblies contained 444 ± 146 contigs and 2.28 × 10^3^ ± 129 predicted ORFs. In total, 2.47 × 10^4^ coding sequences were identified from the assembled contigs, and 1.42 × 10^3^ core and 1.63 × 10^3^ unique accessory genes were identified by comparative genomic analysis of the eleven strains included in this study. After removing genes with biased population distribution, those with redundant patterns of presence and absence, or those presenting problems for subsequent polymerase chain reaction (PCR) primer design, a set of eighty-three candidate accessory genes was identified and used to design an expanded CGF assay aimed at examining the population structure of a large set of *A. butzleri* isolates (n=156) based on shared accessory genome content. Data from eleven accessory genes was discarded due to discordance between *in silico-*predicted CGF profiles and laboratory results on eight isolates sequenced *de novo* as part of this project. The reference CGF-based phylogeny was established from the remaining seventy-two accessory genes.

### A ‘reference phylogeny’ for a sample population of *A. butzleri* isolates

**Figure 1:**
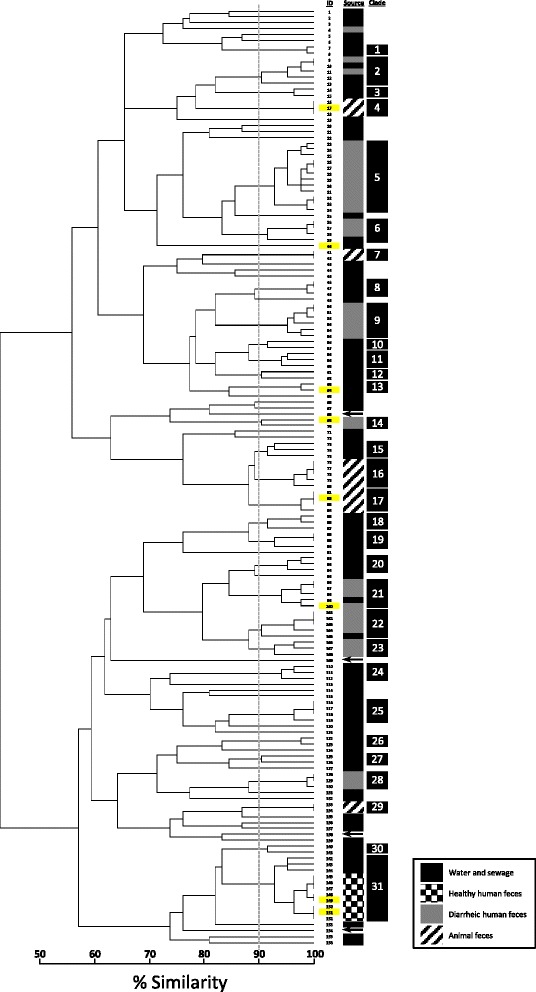
Reference genealogy of *A. butzleri* isolates (*n*=156). Clusters were calculated by simple matching comparison of 72 accessory genes using pairwise coefficients and UPGMA analysis. The scale represents fingerprint similarity based on the total number of shared loci between isolate profiles and the total number of loci in the assay. Dashed grey line represents a 90% similarity threshold used for clade definition. Isolates sequenced as part of this study are highlighted in yellow; ID 17 (strain L353, PRJNA233527), ID 40 (strain L355, PRJNA233527), ID 64 (strain L348, PRJNA233527), ID 69 (strain L352, PRJNA233527), ID 82 (strain L354, PRJNA233527), ID 100 (strain L349, PRJNA233527), ID 149 (strain L351, PRJNA233527), ID 151 (strain L350, PRJNA233527). Published reference *A. butzleri* strains are designated with arrows and include ID 68 (strain 7h1h, PRJNA200766), ID 109 (strain JV22, PRJNA61483), ID 138 (strain RM4018, PRJNA58557), ID 154 (strain ED-1, PRJNA158699).

A reference phylogeny for a comprehensive set of *A. butzleri* isolates (n=156) recovered from river water, raw and treated sewage, diarrheic and non-diarrheic people, and non-human animals was derived from the binary (i.e. presence and absence) data for the expanded CGF assay. The phylogenetic distribution of twelve genome-sequenced strains, which includes four previously sequenced strains and eight strains sequenced as part of this study, shows that all but two strains (149 and 151) belong to distinct CGF clades. Moreover, the *in silico* MLST data (Additional file 1) is consistent with the CGF results because strains 149 and 151 share the same, albeit novel, sequence type while the remaining strains are from diverse sequence types. An average of ten distinct alleles were observed at each of the seven MLST loci, and the lack of shared alleles suggests significant genetic diversity among the twelve WGS strains. Although this dataset does not represent a comprehensive sampling of the *A. butzleri* population, a comparative genomic analysis of these isolates would be expected to capture significant accessory genome diversity. The reference phylogeny contained a total of 31 multi-isolate clades when a ≥90% isolate similarity threshold was applied (Figure 1). The largest clade (Clade 5) comprised 12 isolates from four human diarrheic stool samples. Clade 31 contained all of the isolates recovered from two non-diarrheic human stools. Isolates from non-human animals clustered together and distinctly from other isolates. Although human isolates clustered with water isolates (clades 2 and 31, respectively), there were no clades that contained isolates from both diarrheic and non-diarrheic human beings. None of the four previously sequenced strains included in this dataset clustered at the 90% similarity level with the *A. butzleri* isolates from Southwestern Alberta.

### Analysis of CGF_40_ concordance with reference phylogeny

**Figure 2:**
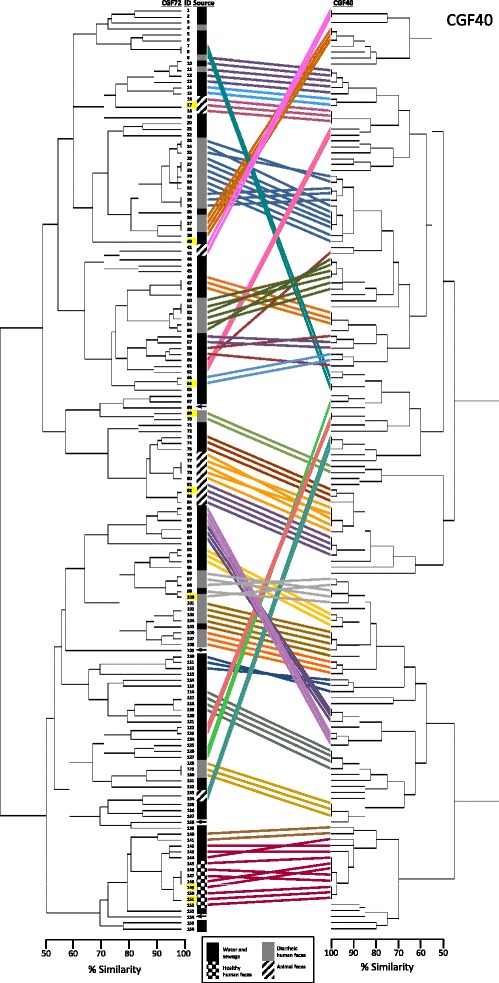
Tanglegram of CGF_72_ (reference) and CGF_40_ genealogies for *A. butzleri* isolates (*n* = 156). Coloured lines represent isolates within clusters in the reference phylogeny that are ≥90% similar to one or more other isolates. Scales represent fingerprint similarity based on the total number of shared loci between isolate profiles and the total number of loci in the assay. Coloured lines also indicate the location of the same isolate in the CGF_72_ and the CGF_40_ cladograms. Scales represent fingerprint similarity based on the total number of shared loci between isolate profiles and the total number of loci in the assay. Isolates sequenced as part of this study are highlighted in yellow; ID 17 (strain L353, PRJNA233527), ID 40 (strain L355, PRJNA233527), ID 64 (strain L348, PRJNA233527), ID 69 (strain L352, PRJNA233527), ID 82 (strain L354, PRJNA233527), ID 100 (strain L349, PRJNA233527), ID 149 (strain L351, PRJNA233527), ID 151 (strain L350, PRJNA233527). Published reference A. butzleri strains are designated with arrows and include ID 68 (strain 7h1h, PRJNA200766), ID 109 (strain JV22, PRJNA61483), ID 138 (strain RM4018, PRJNA58557), ID 154 (strain ED-1, PRJNA158699).

After 1.0 × 10^4^ iterations, CGF Optimizer [25] retrieved 40 accessory genes for CGF_40_ that had an Adjusted Wallace Coefficient (AWC) of 1.0 with respect to the reference phylogeny. Analysis of the 156 *A. butzleri* isolates yielded high Simpson’s ID (Table 1) and AWC (Table 2) values for both assays at 90% and 95% similarity thresholds. In addition, direct comparison showed that clusters in the reference and CGF_40_ phylogenies were highly concordant (Figure 2). At 90% similarity, isolates from 29 of the 31 clades identified in the reference phylogeny also clustered together when analysed using the CGF_40_ assay. Moreover, of the 54 isolates that shared identical CGF_40_ profiles, 45 also shared identical profiles when analysed with the expanded set of 72 markers.

**Table 1:** **Simpson’s Index of Diversity** ^***a***^ **for** ***A. but*** **z** ***leri*** **isolates (** ***n*** **=152) genotyped by CGF**_**40**_

**Partitioning method**	**Assay**	**Partitions** ^*b*^	**Simpson’s ID**	**CI (95%)**	**CINA (95%)**
Binary Pairwise Similarity (UPGMA)	Reference	87	0.984	0.978-0.991	0.977-0.992
	CGF_40_	86	0.987	0.983-0.992	0.982-0.992

^*a*^Simpson’s Index of Diversity (ID), confidence intervals (CI), and non-approximated confidence intervals (CINA) were calculated using the online tool of the Comparing Partitions Website (http://darwin.phyloviz.net/ComparingPartitions/index.php?link=Tool).

^*b*^Partitions were denoted at the 95% similarity level, which was calculated using the simple matching coefficient in BioNumerics (version 6.6, Applied Maths, Austin, TX).

**Table 2:** **Adjusted Wallace Coefficient values** ^***a***^ **of CGF**_**40**_ **compared to the reference phylogeny for** ***A. but*** **z** ***leri*** **isolates (** ***n*** **=152)**

**Partitions** ^**b**^	**Reference (90% Similarity)**	**Reference (95% Similarity)**
**CGF**_**40**_	0.88	0.62
**(90% Similarity)**	(0.83-0.93)	(0.53-0.71)
**CGF**_**40**_	0.92	0.87
**(95% Similarity)**	(0.89-0.95)	(0.83-0.91)

^*a*^Adjusted Wallace Coefficient values were calculated using the online tool of the Comparing Partitions Website (http://darwin.phyloviz.net/ComparingPartitions/index.php?link=Tool).

^*b*^Partitions were denoted by 90% and 95% accessory gene pairwise similarity, which were calculated using the binary simple matching algorithm in BioNumerics (version 6.6, Applied Maths).

### Analysis of CGF_40_ reproducibility

To assess assay reproducibility, the CGF_40_ analysis was repeated for 24 *A. butzleri* isolates on separate occasions. Concordance analysis revealed that 907 of the 920 data points (98.6%) had identical presence/absence patterns in both runs.

## Discussion

Enteritis is inflammation of the alimentary canal (i.e. enteron) that is often characterized by diarrhea, abdominal pain, dehydration, loss of appetite, fever, and nausea [26]. We chose to target Southwestern Alberta as a study site (i.e. a model agro-ecosystem) as this region possesses high rates of enteritis [27], which has been attributed to dense livestock populations in the region [28,29]. *Arcobacter butzleri* is closely related to *C. jejuni* and it is considered by some to be an emerging pathogen [3,7,30] because it has been isolated from diarrheic people [11,31]. However, its pathogenicity and reservoirs/pathways of transmission for potentially pathogenic genotypes have yet to be elucidated. In order to understand the relationship between *A. butzleri* and human illness a method is required for the rapid and accurate genotyping of *A. butzleri* strains to facilitate epidemiological studies.

A number of subtyping methods have recently been used to examine genetic diversity of *Arcobacter* and to compare genotypes between sources [6,30,32]. Douidah *et al*. have recently proposed a two-stage approach using enterobacterial repetitive intergenic consensus PCR and pulsed-field gel electrophoresis for subtyping of human and animal *Arcobacter* isolates [32]. A scheme for MLST, a leading method for related organisms such as *C. jejuni* and *H. pylori*, has recently been developed for *A. butzleri* [6]. No *A. butzleri* sequence types have been directly linked to human illness, but given the relative paucity of data both in the literature and within the global MLST database [33] it is difficult to assess whether the *A. butzleri* MLST data generated so far is representative of large-scale population or epidemiological trends. Moreover, despite the demonstrated ability of MLST to accurately distinguish subtypes of *A. butzleri* and other bacteria, the resources required to generate MLST data for the substantial numbers of *A. butzleri* isolates that are necessary for comparative epidemiological investigations may be prohibitive for many research groups.

Comparative genomic fingerprinting provides a high-resolution and high-throughput alternative to MLST that is also deployable in the context of large-scale epidemiological surveillance [21,22]. The CGF method identifies intraspecies relationships by targeting accessory loci that are representative of genetic variation throughout the genome. The phylogenetic signal in accessory genome content variation has been examined in several bacterial species and was shown to be highly concordant with that contained in other forms of genetic variation ([34-36]). Such loci are binary (i.e. present or absent) and determination of their allelic status does not require sequencing, with assessment possible by PCR amplification. In addition, CGF assays target sufficient loci to distinguish between closely related strains that may be indistinguishable by other methods [21] while generating phylogenetic signal that is consistent with that of MLST [37]. Previous work has shown the CGF assay for *C. jejuni* to be highly predictive of MLST, and although each method clustered strains similarly, CGF provided additional discrimination within those groups [21,22]. In Canada, the CGF method is being used to analyze *C. jejuni* isolates generated through several large-scale surveillance networks, which will facilitate the study of campylobacteriosis through the holistic comparison of *C. jejuni* subtypes collected from a diverse range of sources and infection cases [38].

In addition to being a close phylogenetic relative of *C. jejuni*, two features of the *A. butzleri* pan-genome identified through our comparative genomic analysis suggested that it would be an excellent species for the development of a CGF-based genotyping assay. The *A. butzleri* strains showed significant variability in accessory genome content, which allows for with a high level of discriminatory power when comparing gene carriage. Moreover, we observed that the *A. butzleri* pan-genome contained a significant number of accessory genes, which allowed us to focus on those of greatest genotyping potential. The CGF_40_ assay is based on a marker optimization process that yielded phylogenetic clusters that were highly concordant with those observed in the reference phylogeny, and it provided a high discriminatory power for differentiation of isolates from diverse sources. In addition, the majority of isolates that were identical by CGF_40_ analysis also proved to be identical or highly similar using the larger number of markers. This suggests that the 40 loci that we selected during marker optimization were appropriate for high resolution genotyping of *A. butzleri* strains, and that there may be an “efficiency plateau” above which additional loci do not sufficiently increase discriminatory power to justify their inclusion in the assay. The CGF_40_ assay should be easily deployable, and we found that 32 isolates could be processed (i.e. from stock to digital phylogeny) during a typical workday by a single individual using one thermal cycler and capillary electrophoresis system; more than 1000 *A. butzleri* isolates have been genotyped by one individual in our research group over the course of a 12-month period.

Previous efforts to characterize *A. butzleri* have identified a high degree of genetic variation but have failed to associate specific genotypes in a geographic or temporal context [6,39-41]. In total, we observed 29 clades within the CGF_40_ phylogeny when compared at a similarity threshold of 90% or greater and 121 distinct (i.e. non-identical) CGF_40_ profiles were observed among the 156 isolates analyzed. Of interest, each of the four previously genome-sequenced strains in the public databases formed their own clades in both CGF-based phylogenies. Taken together, these results suggest that the density of marker sampling targeted by the CGF assay described herein provides sufficient power for discriminating isolates at a high level of resolution. At the same time, our observation that 115 of the 156 isolates in this dataset could be assigned to clades with a profile similarity of 90% or greater suggests that this level of discriminatory power does not compromise the ability to identify clades comprised of genetically similar isolates. It is noteworthy that although we developed the CGF_40_ assay with isolates primarily obtained from Southwestern Alberta, the dataset used for the comparative genomic analysis to identify potential CGF markers also included several genome-sequenced isolates from international sources. Moreover, it is our intention to further validate the CGF_40_ assay by examining *A. butzleri* populations in a pan-Canadian and an international context.

*Arcobacter butzleri* were isolated from the stools of diarrheic and non-diarrheic human beings living in Southwestern Alberta, as well as from river and sewage samples throughout Southwestern Alberta during 2008 and 2009. The clustering of isolates from human beings with isolates from river and sewage waters throughout Southwestern Alberta over the same time period suggests that it may be possible for *A. butzleri* strains to be transferred between people and their environment. Although we were unable to identify clades that included isolates from human beings and non-human animals in this dataset, this may be due to the non-concurring sampling of both reservoirs since non-human animal stools were sampled only towards the end of 2009. We hope to examine the possible links between *A. butzleri* found in human beings and animals through concurrent and comprehensive sampling in future studies; the rapid and inexpensive characterization of isolates using our CGF_40_ method for will be very useful in this regard.

To our knowledge no studies conducted to date have examined the carriage and shedding of *A. butzleri* strains in diarrheic and non-diarrheic human beings, and although *Arcobacter* species have been detected in and occasionally isolated from the stools of non-diarrheic individuals [10,11], this is the first time that *A. butzleri* have been isolated from stools of a non-diarrheic human being sampled on two separate occasions. Individuals were sampled six months apart and periodic shedding of the same *A. butzleri* genotype suggests that strains of this bacterium may chronically colonize people without inciting disease. Colonization of healthy human beings by *A. butzleri* may occur in a similar manner to the closely related pathogen *C. jejuni*, which has been shown to colonize healthy people more frequently in areas with endemic rates of infection [23,42,43]. Thus, it may be possible to relate genotypes to endemic disease rates by characterizing *A. butzleri* isolates from diarrheic and non-diarrheic human beings.

## Conclusions

We used WGS and comparative genomic analysis of *A. butzleri* isolated from diverse sources and demonstrated that accessory gene variation among strains can be used for high-throughput, high-resolution, and reproducible subtyping of this bacterium. Although WGS analysis will eventually become the gold standard in epidemiological genotyping of pathogenic bacteria, until WGS data are routinely deployed for surveillance of highly prevalent pathogens, the CGF_40_ assay described herein will allow the scientific community to address key knowledge gaps about the epidemiology of arcobacteriosis toward the prevention and mitigation of enteric disease. Furthermore, the developed CGF_40_ assay is highly deployable and will allow researchers and clinicians to efficiently compare the genetic diversity, persistence, and prevalence of *A. butzleri* subtypes in different sources, and to rapidly and efficiently identify relevant strains as candidates for WGS analysis.

## Methods

### Ethics statement

Scientific and ethics approval to isolate *A. butzleri* from diarrheic and non-diarrheic human beings (i.e. healthy volunteers) was obtained by GDI from the Regional Ethics Committee of the former Chinook Health Region (CHR) and from the University of Lethbridge Human Subject Research Committee. The requirement for informed written consent was waived by the CHR Regional Ethics Committee and the University of Lethbridge Human Subject Research Committee for subsamples of stools submitted by diarrheic people as the samples were submitted for the detection of enteric pathogens at the Chinook Regional Hospital and the identities of patients was not disclosed. Informed written consent as mandated by the University of Lethbridge Human Subject Research Committee was obtained from all healthy volunteers in advance of the submission of stool samples for the isolation of *A. butzleri* and other enteric bacteria.

### *A. butzleri* isolation and DNA extraction

*Arcobacter butzleri* were isolated from one stool sample per person for eleven diarrheic humans, and from two stools obtained from one non-diarrheic human, as well as from non-human animal feces, sewage, and river water collected in Southwestern Alberta during 2008 and 2009. Isolates were streaked for purity and stored at -80°C in Columbia broth (Difco and BBL Microbiology, Lawrence, KS) with 30% glycerol. Isolates from glycerol stocks were grown on Columbia agar (Difco and BBL Microbiology) amended with 10% sheep blood in a microaerobic atmosphere (5% O_2_, 3% H_2_, 10% CO_2_, and 82% N_2_) at 37°C for 24-48 hr, and biomass was collected from the surface of the agar medium. An automated system (Model 740, Autogen, Holliston, MA) was used to extract genomic DNA. Putative *A. butzleri* isolates were identified by PCR amplification using an *Arcobacter* PCR-multiplex assay [9].

### Whole genome sequencing and assembly

For WGS analysis, DNA was extracted using a DNEasy Blood and Tissue Kit (Qiagen Inc, Toronto, ON). To minimize possible genetic bias amongst strains selected for WGS, *A. butzleri* isolates from diverse sources were genotyped using Amplified Fragment Length Polymorphism (AFLP) analysis as described previously [24,44], and eight strains selected to represent highly diverse AFLP profiles were chosen for sequencing (Table 3). The identity of isolate DNA was tested by sequencing approximately 1000 bp of the 16S rRNA gene and by comparing the results with *A. butzleri* sequences within the National Centre for Biotechnology Information (NCBI) genetic database [45,46]. The DNA for isolates to be sequenced was quantified by spectrophotometry (A_600_) (Ultrospec 3100 pro, GE Healthcare Life Sciences, Baie d’Urfe, QC). Isolates were sequenced as paired-end, 100 bp reads on a HiSeq platform (Illumina Inc., San Diego, CA) with Phred30 (99.9%) base-calling accuracy [47], and reads were *de novo* assembled into contigs using ABySS [48] with specifications for short paired-end reads. Sequencing data for the *A. butzleri* isolates were accessioned in the NCBI genetic sequence database as a single bioproject (PRJNA233527).

**Table 3:** **Isolates of** ***A. but*** **z** ***leri*** **from diverse sources for whole genome sequence analysis**

**Strain**	**Source**	**Location**	**Collection date**
L348	Sewage outfall	Lethbridge, Alberta, CA	07/May/2008
L349	Diarrheic human stool	Blairmore, Alberta, CA	30/Jul/2008
L350	Non-diarrheic human stool	Lethbridge, Alberta, CA	30/Sep/2008
L351	Non-diarrheic human stool	Lethbridge, Alberta, CA	01/Apr/2009
L352	Diarrheic human stool	Lethbridge, Alberta, CA	27/Apr/2009
L353	Horse feces	Diamond City, Alberta, CA	02/Jul/2009
L354	Pig feces	Lethbridge, Alberta, CA	12/Aug/2009
L355	Raw sewage	Lethbridge, Alberta, CA	08/Mar/2009

### Detection and identification of coding sequences

Rapid Annotation Using Subsystem Technology [49] was used to identify open reading frames (ORF) for the eight sequenced *A. butzleri* genomes, as well as three previously available genome assemblies (RM4018 - PRJNA58557, ED1 - PRJNA158699, JV22 - PRJNA61483). The genome assembly for a fourth strain, 7h1h (PRJNA200766), was not available at the time that the comparative genomic analysis was performed, however we were able to utilize the four published WGS strains for all subsequent *in silico* CGF analyses.

To identify core and accessory genes, the ORFs from each genome were searched against the eleven genome assemblies using the program BLASTP from the Basic Local Alignment Search Tool [45,46], with filtering to remove redundant results from likely orthologous genes. ORFs present in all assemblies were identified as core, and all non-redundant ORFs absent from one or more strains were designated as accessory.

### Identification of candidate accessory genes for CGF assay development

To simplify CGF assay design, accessory genes with limited genotypic potential due to a highly biased population distribution (i.e. present in greater than 80% of strains or present in fewer than 20% of strains) were eliminated from further consideration as candidate markers. Moreover, for groups of accessory genes that presented redundant patterns of presence and absence in the dataset (i.e. genes that are typically linked and provide limited additional discrimination), only one representative gene from each unique pattern was considered as a candidate marker for CGF development. Short genes (i.e. <300 bp) and/or those containing nucleotide gaps or polymorphisms that might affect PCR primer design were also discarded. Accessory genes meeting the above criteria were identified and used to design an expanded CGF assay (i.e. the reference assay) to examine the population structure of a diverse collection of *A. butzleri* isolates (n=152) based on accessory genome variability. Data from these isolates, which were recovered from river water, raw and treated sewage, diarrheic and non-diarrheic human beings, and non-human animals in Southwestern Alberta was used in conjunction with *in silico-*derived [50] CGF data from four published genome-sequenced strains (RM4018 - PRJNA58557, ED1 - PRJNA158699, JV22 - PRJNA61483, 7h1h - PRJNA200766). CGF profiles were also generated *in silico* using the program MIST [50] for the eight isolates sequenced *de novo* to allow for comparison with PCR-derived CGF data, thus facilitating assessment of marker performance. A dendrogram representing an estimate for a ‘reference phylogeny’ was constructed from the binary (i.e. presence and absence) data for those genes that generated data fully concordant with *in silico-*predicted CGF profiles (n=72). Hierarchical clustering was performed by the unweighted pair group method with arithmetic mean (UPGMA) using the *hclust* function in R [51] and the simple matching coefficient of genetic similarity.

### Optimization of markers for development of final CGF assay

The program CGF Optimizer [25], which calculates the AWC and the Robinson-Foulds Symmetric Distance (SymD) [52-55] to assess the concordance between clustering results from sets of prospective CGF markers and a reference phylogeny, was used to identify a subset of accessory genes yielding high concordance to the reference phylogeny generated using the expanded CGF assay. Briefly, CGF Optimizer was used to subsample sets of candidate accessory genes and to compute the AWC of each set to the reference phylogeny; the 40 loci that were most concordant with the reference phylogeny (i.e. the set with the highest AWC) were selected for the final CGF_40_ assay.

### CGF assay development

Primer3 [56] was employed to design PCR primers for genes selected for CGF assays (Additional file 2). The programs MultiPLX [57] and CGF Multiplexer [25] were used to arrange primers with compatible thermodynamic properties into multiplex pools that would generate amplicons differing by at least 100 bp to facilitate unambiguous scoring of marker presence or absence. The CGF profiles obtained *in silico* [50] and by multiplex PCR amplification for the sequenced strains were compared to ascertain primer sensitivity and specificity, and primer pair concentrations within each multiplex were adjusted to optimize product amplification (Table 4). In addition, the reproducibility of the final CGF_40_ assay was tested by running duplicate PCR reactions for a set of 24 *A. butzleri* isolates (23 test isolates plus 1 control). To generate a CGF profile, eight PCR reactions targeting five loci per reaction were performed for each *A. butzleri* isolate. Individual PCR reactions (25 μl) contained 2.0 μl of genomic DNA, 2.5 μl of 10X incubation mix without MgCl_2_ (MP Biomedicals, Solon, OH; 1X), 2.5 μl of MgCl_2_ (MP Biomedicals; 2.5 mM), 0.5 μl of a deoxynucleoside triphosphate pool (0.2 mM), 1.0 μl of the multiplex primer pool (0.4 μM), 0.2 μl Taq DNA Polymerase (MP Biomedicals; 1 U μl^−1^), and 16.3 μl Optima water (Fisher Scientific, Ottawa, ON). PCR conditions consisted of 32 cycles of denaturation at 93°C for 30 s, annealing at 60°C for 90 s, and extension at 72°C for 60 s. After a final extension step at 72°C for 5 min, PCR products were stored at 4°C, and visualized using a QIAxcel automated capillary electrophoresis system (Qiagen Inc.) with a QIAxcel 2400 Sample DNA Screening Kit (Qiagen Inc.), QX 15-1000 bp alignment marker (Qiagen Inc.), and 30 ng μL^−1^ QX 50-800 bp Size Marker (Qiagen Inc.). Capillary electrophoresis lanes were scored for amplification of the five loci targeted (i.e. scored as present or absent) in each multiplex PCR, resulting in a 40-digit binary profile for each isolate. Isolate profiles were clustered using the simple matching coefficient in BioNumerics (version 6.6, Applied Maths, Austin, TX), and isolate similarity was visualized as an UPGMA dendrogram.

**Table 4:** **Primers for PCR amplification of CGF**_**40**_ **markers** ^***a***^

	**Product siz** **e (bp)**	**Primer forward (5′ to 3′)**	**Primer reverse (5′ to 3′)**	**Concentration** ^***b***^ **(μM)**
Multiplex 1	150	GCATCCTCTTCCTCCATCAT	TCGAATAAATCCCCTACCCTT	12
	250	ATACACCACCAGATGAGCTG	TAACGTACCGCATCCATTGA	10
	400	AGTGCCCGTTCTATTGGTAT	GCATAAAGAGCTTCTCCTCC	8
	500	ACTCTTCCCGAATCTGCAAT	TCTCCAATTCCTTGTCCTATTGT	10
	600	AGTCATGCAATCCTAACGAGA	AGGAGCCTACTATGTACCTCT	10
Multiplex 2	150	TTTTCATTGGGAAGAAGAATTTAGT	TCCAATTCATAAATATCTCTTGGTGA	12
	250	TCTTTTAAAGAAGACAGCTGTAGT	TTTTGCAACACCTAATCTTGC	18
	350	TGATACAGGAATTATAAGAAGTGTTCC	GCATGAACTTCAACTCCAGG	5
	450	TGGAAATGACAGAGGATGGT	AGTAACGGATGAGCTTTTAAATTT	8
	600	TTGGGCTATTATGTCCCCAG	TCGTACAACTGGCATAGCTT	7
Multiplex 3	200	CCTCAACTTCTAACAGCAGG	CTCACATCACCCAATCCACT	8
	300	TGGAATATCATAAACCAAAAATTGTTT	TTCATTGCAAATCCGCCTTT	10
	450	ACAGCATCCTTGATTCTAGCA	GTGTAATCATAGCCCAAATCCA	12
	550	TGAAATAATGAATGAACACAATAGCA	GTGCACAACCTAAAACCTCA	10
	700	GACAGGAACAGAGGGAAGTC	AGCATCTTTATTTGTCGCACT	10
Multiplex 4	200	TGATGAAACACTAGAAAATAAGGCT	CCAGTAAAACCTCTGTCAGC	11
	350	TCACTTTTAGGTACTCACGACT	GCTATAAAACTTGCACCTTTATCG	9
	450	CAAAGATTTCTACGGGAAATTTGT	ACATCCTTTGCCTCTTTAAAAGA	9
	550	TCGAGGACAAGCAGATTCAA	GCCATTTCTACTTCCATTGTGT	7
	700	ACAGCAGTAACATTACAGGG	TCAAAAGCAATTCCACCACT	11
Multiplex 5	150	TCTATAGGTGCTGACCCACT	GCCGCAATACTTCCAAAACT	9
	250	TTTACAGGAGCTTGGACATCA	TTTTACCATCATCTTCAACCCA	9
	400	CATCGTCCTTCAGTCGAATAT	GGAAACCATTTTCTTTTGCCA	9
	550	GTCATTTTTACACCACCTGCA	TCAAAACGCTTAGCCAAATCT	12
	700	ACTTTTTGCTTCTCAAAGTAGAAC	CCTCTGAAAAATTGAAATAATATACCC	10
Multiplex 6	150	GGTTGGGGAAAACTGCTTTT	TCTCTTGATTTTTAGTTTCAATCTCT	10
	250	TGCTATGGGTGCAATGGTTA	AAGATTCTAGCAACACCCGA	8
	400	TGGGGACATGAAAACTGGAA	TTCACATACTTTCTCAGGCATT	10
	550	ACTATGGCTATATATGCGAAGAAA	TCCATAAATGTTTCAACTCAGGA	10
	650	GGAATTGCCGAGTTTACACG	TGAGCTCCATGTTGTATTGGA	10
Multiplex 7	200	ACTCCATTTGTGCTTATTGGA	TCTTGAACTAGCCAAAAGTGC	10
	350	TCGAAATATCTTTTAGCTTCAAGAA	AAAACATCATTTTCTTTTGCCCA	10
	450	AGAGTTTGGATGGAAAACTGT	TGCAACTATTCCATCAAAACCA	10
	550	GGTTCAACACCAGGAACAAA	TGCAACACCTATCATCTCATTT	10
	700	GGAAAAGGCAAAGAATCCTCA	ACCATCGCCAGACTTCATTA	10
Multiplex 8	150	TGCAAGAAATGGTGGAACAA	CCTGTTGCAATAGTTGGTGT	10
	250	TGGTAGAAGAAACAATAAAAAGATTTG	AGTCTTGATTTATCGACAGTTCT	10
	350	TTTTGTTTGAAGCTTATTCGTGA	AGTCCATATCCTTTCTCTCTCA	8
	450	AGGAGCTGTTGAGATTTTCAA	GTCGTTGCTCATCTGCTTTT	7
	550	GATGCTGGATTTTGTATGGCT	AGCCAAGAAACTTTCAATATCTCT	10

^*a*^Primer pairs were selected and grouped into multiplexes using Primer3 [56], multiPLX [57], and CGF Multiplexer [25].

^*b*^Multiplex primer pair concentrations were optimised for T_a_ = 60°C.

### Assessment of CGF discrimination and concordance

PCR data for the reference and CGF_40_ assays was generated for the 152 *A. butzleri* isolates. The CGF profiles of four previously published genome-sequenced strains (RM4018, ED1, JV22, and 7h1h) were also obtained *in silico* [50]. To verify concordance between the expanded CGF and CGF_40_ assays, binary data from each assay was subjected to hierarchical clustering by UPGMA using the *hclust* function in R [51] and the simple matching coefficient of genetic similarity. The online ‘Comparing Partitions’ tool [52] was used to calculate the discriminatory power of each assay and the concordance between assays. The discriminatory power of each CGF assay was calculated using Simpson’s ID [58], and the concordance was calculated as the AWC value between the CGF_40_ assay and the reference phylogeny. A “tanglegram” was generated using a custom R script to compare dendrograms for the CGF_40_ and the reference phylogeny. This script is available online at https://gist.github.com/peterk87/d92f81ae475063792f49. Briefly, the script generates the dendrograms from binary CGF_40_ and reference phylogeny data and rearranges the CGF_40_ dendrogram with respect to the reference phylogeny in order to maximize structural concordance or minimize entanglement of branches using the “untangle_step_rotate_1side” function from the R package *dendextend* (https://github.com/talgalili/dendextend). It then uses the reference phylogeny to create color-coded linkage groups at a 90% cluster similarity level and plots the color-coded tanglegram.

## Additional files

Additional file 1:
**Cluster analysis of** ***A. butzleri*** **strains** **(** ***n*** **=12) by multilocus sequence typing, amplified fragment length polymorphism, and comparative genomic fingerprinting.**
Additional file 2:
**Identification of genes targeted by primer pairs in CGF**_**40**_ **multiplex PCR.**
